# Effectiveness of radioiodine therapy on preventing recurrence in differentiated thyroid carcinoma: a systematic review

**DOI:** 10.1186/s43046-025-00293-z

**Published:** 2025-05-21

**Authors:** Bima Indra, Nur Qodir, Didit Pramudhito, Legiran Legiran, Zen Hafy, Andi M Iqbal Yusran

**Affiliations:** 1 Faculty of Medicine, University of Sriwijaya, Palembang, Indonesia; 2 Department of Surgery, University of Sriwijaya/ Mohammad Hoesin General Hospital, Palembang, Indonesia; 3 Department of Biomedicine, University of Sriwijaya, Palembang, Indonesia; 4 Nuclear Medicine Division, Department of Oncology, Mohammad Hoesin General Hospital, Palembang, Indonesia

**Keywords:** Differentiated thyroid carcinoma, Radioiodine therapy, Thyroglobulin, Papillary thyroid carcinoma

## Abstract

**Background:**

The effectiveness of radioiodine therapy (RAI) in reducing recurrence and improving overall survival in differentiated thyroid carcinoma (DTC) remains debated. This systematic review evaluates the impact of RAI on DTC recurrence and survival.

**Methods:**

A comprehensive search was conducted across PubMed, ScienceDirect, Web of Science, CINAHL, and Tripdatabase, including studies from inception to August 2024. Only studies published in English with full-text availability were included. Risk of bias was assessed using the Revised Risk of Bias Assessment Tool for Nonrandomized Studies of Interventions (RoBANS 2).

**Results:**

Nine studies were included, involving 161,703 participants (36,658 men and 125,045 women). The studies were geographically diverse, with four from the American continent, three from Asia, and two from Europe. RAI doses ranged from 30 to 300 mCi, with 30 mCi and 100 mCi being the most common. Five studies found that RAI reduced recurrence, while two found no significant effect. The median time to recurrence ranged from 10 months to 15 years, with most studies indicating a 1–2-year median. Regarding overall survival, two studies reported improvement with successful RAI therapy, while two found no significant impact.

**Conclusion:**

RAI therapy shows potential in reducing recurrence in DTC, particularly within the first 2-year post-treatment, but its effect on overall survival remains unclear. Further high-quality research is necessary to confirm these findings and guide clinical practice.

## Introduction

Differentiated thyroid carcinoma (DTC) is the most common form of thyroid cancer, accounting for approximately 90% of all thyroid cancer cases in developed countries [1]. DTC originates from follicular cells and includes subtypes such as papillary thyroid carcinoma (PTC) and follicular thyroid carcinoma (FTC) [2]. PTC is particularly prevalent, representing around 80% of all thyroid tumors [3]. The management of DTC primarily focuses on strategies to prevent disease recurrence, utilizing imaging, serum thyroglobulin monitoring, thyroid hormone therapy, and targeted therapies [4]. Generally, DTC is associated with a favorable prognosis, especially when diagnosed early and managed appropriately, with most patients responding well to standard treatments [5, 6]. However, certain factors, such as distant metastases, extrathyroidal invasion, high-grade tumors, and older age, may increase the risk of recurrence and mortality in DTC patients [5].

Radioactive iodine (RAI) therapy, particularly with iodine-131, is a cornerstone in the treatment of DTC. It plays a crucial role in eliminating residual thyroid tissue and targeting any remaining cancer cells after thyroidectomy [7]. The efficacy of RAI therapy is largely due to the ability of thyroid cells to concentrate and retain iodine, facilitated by the sodium-iodide symporter (NIS), which allows for targeted radiation delivery to malignant thyroid tissue while minimizing systemic effects [8, 9]. Over the years, advancements in RAI therapy have refined dosing strategies and treatment protocols, contributing to its role in improving survival rates and reducing recurrence in DTC patients, particularly those with certain risk factors or iodine-avid distant metastases [10, 11].

It is important to consider prior studies that have significantly contributed to the understanding of RAI therapy in DTC. For instance, a study from Wang et al. (2020) demonstrated that RAI ablation provides clear benefits in reducing recurrence in intermediate-risk papillary thyroid cancer, reinforcing its value in specific risk groups. [12] Other studies provided long-term, institution-based analyses, highlighting prognostic factors and treatment outcomes over several decades, including the roles of surgery, RAI, and external beam radiation [13, 14]. These foundational studies support the view that patient selection based on risk stratification is key to maximizing RAI benefit. Additionally, recent evidence from Toraih et al. (2024) focused on the pediatric population, showing that RAI ablation significantly reduces the risk of recurrence in children with DTC. [15]

Despite its established role, the effectiveness of RAI therapy in reducing recurrence and enhancing overall survival in DTC remains a topic of debate. While some studies support its benefits, others have reported inconsistent findings, particularly concerning the long-term impact of RAI on overall survival and disease control [8]. The potential risks associated with internal radiation exposure from RAI, such as the development of secondary malignancies, have also been a concern [16]. Furthermore, the management of DTC with aggressive therapies, including RAI, has sparked discussions regarding the balance between the benefits and potential risks, leading to conflicting recommendations in clinical practice [17]. These discrepancies may stem from variations in study design, patient characteristics, RAI dosages, and follow-up durations. Given these inconsistencies, there is a critical need to systematically review and synthesize the available evidence. This systematic review aims to provide a comprehensive evaluation of the impact of RAI on recurrence in DTC patients, helping to clarify its role in clinical practice and identify areas where further research is needed. By addressing these gaps in the literature, the review seeks to offer clearer guidance for clinicians in optimizing the use of RAI for managing DTC.

## Methods

This systematic review followed a PRISMA guideline to evaluate the impact of radioiodine therapy on the recurrence of differentiated thyroid carcinoma [18]. Three authors independently conducted an extensive literature search across five electronic databases: PubMed, ScienceDirect, Web of Science, CINAHL, and Tripdatabase. The search included studies from the inception of each database up to August 2024. The search strategy combined relevant keywords and Medical Subject Headings (MeSH) terms related to “radioiodine therapy,” “differentiated thyroid carcinoma,” “papillary thyroid carcinoma,” and “recurrence.” Boolean operators (AND, OR) were used to refine the search, ensuring the inclusion of all pertinent studies.

Studies were selected based on the following criteria: it discussed the effect of radioiodine therapy on differentiated thyroid carcinoma recurrence, were published in English, and had full-text availability. Eligibility criteria were shown in Table 1. Titles and abstracts were initially reviewed, followed by a full-text assessment of potentially relevant articles. Any disagreements between the three reviewers during the selection process were resolved through discussion with a fourth independent reviewer. The included studies demonstrated substantial heterogeneity in patient characteristics, radioiodine dosages, and study designs, which prevented the performance of a meta-analysis. This variability may have affected the overall findings and highlights the difficulty in reaching definitive conclusions.

**Table 1:** Eligibility criteria for this review

Criteria	Inclusion	Exclusion
Population	Patients with differentiated thyroid carcinoma	Patients with other types of thyroid cancer
Intervention	Radioiodine therapy	Studies not involving RAI treatment
Outcome	Recurrence of DTC, recurrence-free survival (RFS), overall survival (OS), thyroglobulin (Tg) levels	Studies not reporting recurrence, survival, or Tg outcomes
Study design	Randomized controlled trials (RCTs), cohort studies, case–control studies, observational studies	Case reports, case series, editorials, reviews, conference abstracts
Language	English	Non-English studies
Full-text availability	Full-text accessible	Studies without full-text availability
Publication date	From database inception to August 2024	-

To ensure the quality and reliability of the included studies, the risk of bias was assessed using the Revised Risk of Bias Assessment Tool for Nonrandomized Studies of Interventions (RoBANS 2) [19]. The three authors independently conducted the risk-of-bias assessment, with discrepancies resolved through discussion among all authors. References were organized using Mendeley Reference Manager to maintain accuracy and consistency in citation management.

## Results

**Fig. 1:**
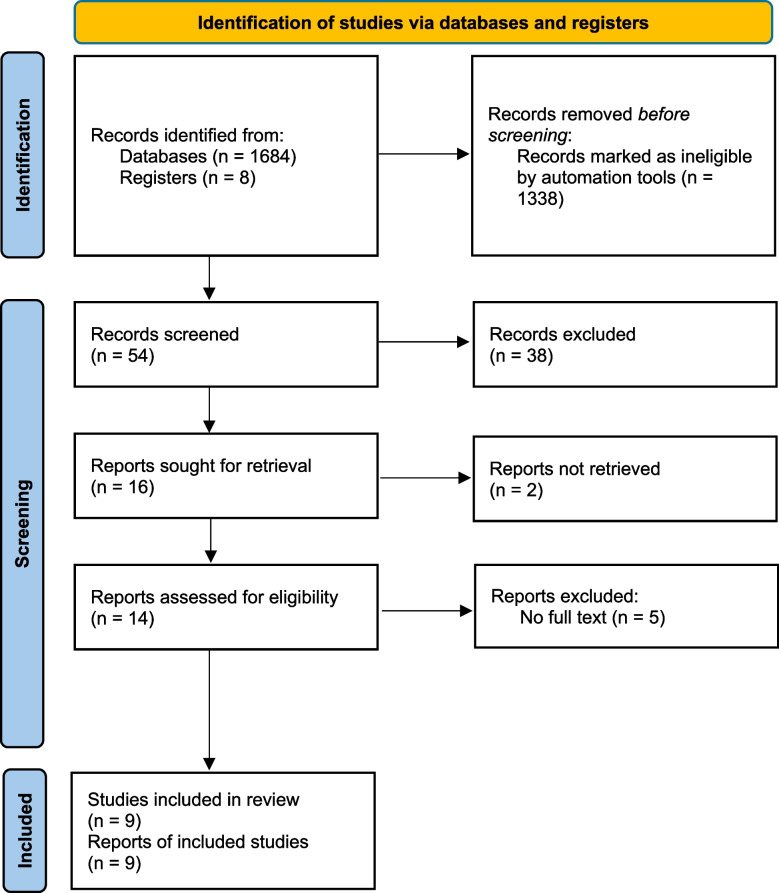
PRISMA flowchart

The search strategy involved querying five electronic databases, including PubMed, ScienceDirect, Web of Science, CINAHL, and Tripdatabase, resulting in 1692 articles (651 from PubMed, 1013 from ScienceDirect, and 28 from other databases). The initial article-type screening reduced this to 54 articles, which were further narrowed to 14 articles after screening based on the article title. Full-text availability and relevance were then assessed, leading to the exclusion of five articles that did not meet the criteria [20–24]. Ultimately, nine studies were included in the final review, ensuring a thorough and systematic approach to evaluating the impact of radioiodine therapy on differentiated thyroid carcinoma recurrence. The search process is shown in Fig. 1.

A total of nine studies were included in this systematic review. The geographic distribution of the studies was as follows: four studies from the American continent, three from Asia, and two from Europe. Regarding the risk of bias, four studies were assessed as having a high risk of bias, two had a medium risk, and three were determined to have a low risk of bias. Specific concerns were noted in four studies related to the comparability of the target group domain, while three studies had issues concerning target group selection and confounding factors. No significant concerns were found in the domains of exposure measurement, blinding of assessors, outcome assessment, incomplete outcome data, or selective outcome reporting. The risk-of-bias assessment is shown in Table 2.

**Table 2:** Risk-of-bias assessment using ROBANS-2

No	Domain	Study index number								
		**1**	**2**	**3**	**4**	**5**	**6**	**7**	**8**	**9**
1	Comparability of the target group	Low	Low	Low	Low	High	High	High	High	Low
2	Target group selection	Low	Low	High	Low	High	High	Low	Low	Low
3	Confounders	Low	High	High	Low	Low	Low	Low	High	Low
4	Measurement of exposure	Low	Low	Low	Low	Low	Low	Low	Low	Low
5	Blinding of assessors	Low	Low	Low	Low	Low	Low	Low	Low	Low
6	Outcome assessment	Low	Low	Low	Low	Low	Low	Low	Low	Low
7	Incomplete outcome data	Low	Low	Low	Low	Low	Low	Low	Low	Low
8	Selective outcome reporting	Low	Low	Low	Low	Low	Low	Low	Low	Low
Overall results		Low	Medium	High	Low	High	High	Medium	High	Low

The nine studies included in this review encompassed a total of 161,703 participants, with 36,658 men and 125,045 women. The mean age of participants ranged from 44 to 55.2 years, with the youngest participant being 9 years old and the oldest 89 years old. Six studies focused on patients with PTC, while three included patients with DTC more broadly. The dose of RAI administered varied between 30 and 300 mCi, with 30 mCi and 100 mCi being the most used doses.

While our inclusion criteria focused on studies examining the impact of RAI therapy on DTC outcomes, not all studies reported both recurrence and overall survival. Out of the nine included studies, seven provided data on recurrence rates, and only four reported overall survival outcomes. Therefore, when synthesizing the results, analyses related to recurrence were based on the seven studies that reported these data, and the assessment of overall survival was derived from the four studies that included such outcomes.

In terms of outcomes, five studies concluded that RAI therapy could reduce the recurrence of thyroid carcinoma, while two studies reported findings to the contrary. The median time to recurrence ranged from 10 months to 15 years, with most studies indicating a median time to recurrence of approximately 1 to 2 years. Several variables were identified that influenced the success of RAI in preventing recurrence. Factors such as older age, high thyroglobulin (Tg) levels before RAI therapy, and extranodal extension were associated with less successful outcomes. Conversely, successful therapeutic response, higher RAI doses, and the presence of the classic type of PTC were associated with a lower risk of recurrence.

Among the studies included in this review, three studies demonstrated that RAI therapy significantly reduced Tg levels, indicating a positive response to treatment and effective ablation of thyroid tissue. These studies showed a consistent decrease in Tg levels following RAI, suggesting its potential role in reducing tumor burden and aiding in the monitoring of differentiated thyroid carcinoma (DTC) recurrence. However, one study reported contradictory findings, showing no significant decrease in Tg levels after RAI treatment. Regarding overall survival, two studies suggested that successful RAI therapy could improve survival rates, whereas two studies did not find a significant impact on overall survival. A summary of the included studies is presented in Tables 3 and 4.

**Table 3:** Summary of the included studies

No	Study detail	Sample detail	Outcome
1	Iizuka, Yusuke et al. [25] Japan, 2023 Retrospective study	*N* = 284 patients who received RAI therapy (low dose 30 mCi or high dose 80–100 mCi) in the hospital for DTC after surgical resection without macroscopic residual or metastatic lesions Men:women = 92:192 Median age = 54 years (9–85)	• Successful radioiodine therapy is associated with lower recurrence and death in patients with thyroid carcinoma • The 3‑year recurrence-free survival rate (RFS) was 96.3% in the group with successful RAI compared to only 66.1% with failed RAI • Histology type (except for papillary carcinoma) and Tg level > 4 ng/dL before radioiodine therapy significantly exacerbated the RFS rate
2	Cadena-Pineros, Enriques et al. [26] Colombia, 2022 Retrospective study	*N* = 48 PTC patients who received total thyroidectomy, RAI remnant ablation, and surgery to remove the locally recurrent/persistent PTC and received a second RAI therapy (100–200 mCi, mean 102 mCi) Men:women = 10:38 Median age = 47.03 years (21–73)	• 77.1% of the patients did not have another recurrence. The median relapse-free time from the second RAI treatment was 10.9 months (range: 1.3–58.2 months) • TSH levels decreased after surgery and iodine treatment, although statistically not significant • Tg levels dropped after surgery and declined after a second RAI treatment, but the changes were not statistically significant • TgAb levels significantly decreased at each stage, showing statistical significance
3	Dang, Dung Trung et al. [27] Vietnam, 2022 Retrospective study	*N* = 71 patients with histologically confirmed PTC were initially treated by thyroidectomy, with or without lymph node dissection and with either remnant ablation or adjuvant RAI treatment Men:women = 7:64 Mean age = 45 years (15–74)	• The median number of radioactive iodine therapy courses before RAI-R PTC was diagnosed two times (at least one time, at most nine times), with a median dose of 300 mCi • The median time to recurrence was 27 months (8–117). The 1- and 2-year PFS rates were 85% and 74.6%, respectively
4	Holoubek, Simon A. et al. [28] United States, 2021 Retrospective study	*N* = 160,458 patients with PTC (155,940 classic PTC, 4011 tall cell PTC, and 507 diffuse sclerosing PTC) with 1- to 40-mm tumor size Men:women = 36,340:124,118 Mean age = 49 years	• RAI on average was used on 62% of the aggressive variant population compared with only 43% of those with classic PTC • Overall survival was improved in the total thyroidectomy with RAI patients with tumor sizes 2.1 to 4.0 cm compared to those undergoing total thyroidectomy alone (82.4% vs 80.8%, P ¼ 0.027) • There were no differences in 10-year overall survival between the patients who received total thyroidectomy without RAI versus total thyroidectomy with RAI for tumors ≤ 2 cm • Not having RAI is a predictor of decreased overall survival
5	Bouvet, Clement et al. [29] France, 2019 Retrospective study	*N* = 85 patients treated for DTC presented with locoregional (cervical) cancer persistence or recurrence after initial thyroidectomy and RAI (100 mCi) for remnant ablation were included Men:women = 26:59 Mean age = 50.5 years	• Papillary cancer was the predominant histological type of carcinoma (92.9%) • Disease recurrence after re-treatment was detected in 31 patients (36.5%), with a median recurrence-free survival of 15.9 years • Disease remission rates in the adjuvant RAI and follow-up groups did not differ (61 vs. 69%) • Multivariate analyses and Kaplan–Meier curves did not show any significant benefit of ad-RAI in patients with poor prognostic factors (age > 55 years) • Patients older than 55 years have a higher risk of recurrence independently of time, employment, or not of adjuvant RAI
6	Gray, Katherine D. et al. [30] France, 2018	*N* = 183 patients undergoing RAI treatment for high-risk PTC (based on ATA 2015 criteria) Men:women = 49:134 Mean age = 47 years	• Patients in the intermediate-dose (100 mCi) RAI group had a significantly higher rate of recurrence than patients in the high-dose (150 mCi) RAI group • Time to recurrence was shorter in the intermediate-dose group than in the high-dose group, with a median of 0.8 years vs a median of 2.5 years • The lymph node metastases and local recurrence rate were higher in the intermediate-dose group • There was no difference in the incidence of recurrence in the form of distant metastases between groups • The only independent predictor of recurrence was extranodal extension
7	Hung, Matthew L. et al. [31] United States, 2018 Retrospective study	*N* = 102 patients with PTC who had undergone initial total thyroidectomy with or without subsequent RAI ablation and then had a reoperation for locoregional recurrence Men:women = 35:67 Median age = 44 years (33–54)	• Median dose of RAI was 150 mCi • Patients who received RAI after reoperation had outcomes like those of patients who underwent reoperation alone • Recurrence after reoperation occurred in 18 of 50 patients (36%) in the reoperation with RAI group and 10 of 52 patients (19%) in the reoperation without RAI group • Receipt of RAI after reoperation was not associated with the rate of a second structural recurrence • In the reoperation without RAI group, more patients had an excellent response based on Tg levels compared to the RAI group
8	Mujammami, Muhammad et al. [32] Canada, 2016 Observational study	*N* = 370 PTC patients with total thyroidectomy and received 30-mCi radioiodine for remnant ablation Men:women = 68:302 Mean age = 55.2 years (25–89)	• The response at 12 to 18 months to 30-mCi remnant ablation was excellent (negative imaging and suppressed Tg < 0.2 ug/L or stimulated Tg < 1.0 µg/L) in 79.3% of patients in the THW group and 76.0% of the rhTSH group • Variables that were significantly associated with remission in the univariate analysis included classical-type PTC (*p* < 0.001), the absence of extrathyroidal extension (*p* < 0.006), and an excellent response to therapy (*p* < 0.001) • Excellent response to radioiodine at 12 to 18 months correlated significantly with long-term remission rates
9	Zhang, Yingjie et al. [33] China, 2015 Randomized study	*N* = 102 patients that had undergone total thyroidectomy with histologically confirmed DTC; pT4 stage (with extrathyroidal extension according to both operation record and pathological report) and any N stage, with no evidence of DM; had ps-Tg ≤ 5 ng/ml and thyroglobulin antibodies level ≤ 46 IU/ml; and had no iodine contamination Men:women = 31:71 Age = 62.7% of patients < 45 years	• No patient had clinical recurrences during the mean 6.8 months of follow-up • No significant difference in success rate between the low-dose (30 mCi) and high-dose (100 mCi) groups • RAI success rate was 96% in the low-dose group and 98% in the high-dose group based on ^131^I whole-body scan and stimulated Tg

*DTC* Differentiated thyroid carcinoma, *PTC* Papillary thyroid carcinoma, *RAI* Radioactive iodine, *RFS* Recurrence-free survival, *rhTSH* Recombinant human thyrotropin, *Tg* Thyroglobulin, *TgAb* Thyroglobulin antibody, *TSH* Thyroid-stimulating hormone

**Table 4:** Summary of the key finding

Study ID	RAI dose details	Follow-up/recurrence outcomes	Key findings & variables
1	Low dose: 30 mCi; high dose: 80–100 mCi	Three-year RFS: 96.3% with successful RAI vs. 66.1% with failed RAI	Non-PTC histology and Tg > 4 ng/dL before RAI were associated with poorer RFS
2	100–200 mCi (mean 102 mCi)	77.1% of patients remained recurrence-free; median relapse-free time: 10.9 months	TSH and Tg levels decreased post-treatment (Tg reduction not statistically significant), while TgAb levels significantly decreased
3	Median dose: 300 mCi (multiple courses, 1–9 sessions)	Median time to recurrence: 27 months; 1-year PFS: 85%, 2-year PFS: 74.6%	Demonstrated benefit with repeated RAI courses prior to diagnosis of RAI-refractory PTC
4	Not specified; RAI usage: 62% in aggressive variants vs. 43% in classic PTC	Improved overall survival (OS) in tumors 2.1–4.0 cm (82.4% vs. 80.8%); no difference for tumors < 2 cm	Not having RAI is a predictor of decreased overall survival; benefit is seen in larger tumors
5	100 mCi	36.5% experienced recurrence; median RFS: 15.9 years	Patients older than 55 years had a higher risk of recurrence regardless of adjuvant RAI
6	Intermediate dose: 100 mCi; high-dose: 150 mCi	Median time to recurrence: 0.8 years (intermediate) vs. 2.5 years (high)	Extranodal extension was the only independent predictor of recurrence, the lymph node metastases and local recurrence rate were higher in the intermediate-dose group, and higher doses were associated with longer recurrence-free intervals
7	Median dose: 150 mCi	Recurrence after reoperation: 36% in the group with RAI vs. 19% without RAI	Post-reoperation RAI did not reduce second structural recurrences; non-RAI group showed better Tg response
8	30 mCi for remnant ablation	Excellent response at 12–18 months in 79.3% (THW) and 76.0% (rhTSH) groups	Classical-type PTC and the absence of extrathyroidal extension were significantly associated with remission
9	Comparison: low dose (30 mCi) vs. high dose (100 mCi)	No clinical recurrence over a mean follow-up of 6.8 months; success rate: 96% (low dose) vs. 98% (high dose)	No significant difference in outcomes between low and high doses

## Discussion

This review synthesized data from nine studies to evaluate the impact of RAI on the recurrence and overall survival of patients with DTC. The findings revealed that while most studies (five out of nine) supported the effectiveness of RAI in reducing the recurrence of thyroid carcinoma, there were also studies that found no significant impact on overall survival. The median time to recurrence varied widely across studies, ranging from 10 months to 15 years, with most recurrences occurring within the first 1- to 2-year post-treatment. These results highlight the potential of RAI therapy to play a critical role in the early management of DTC, particularly in reducing the risk of recurrence. The risk-of-bias assessment using RoBANS 2 revealed variability across the included studies, with several studies rated as high risk (studies 3, 5, 6, and 8), some as medium (studies 2 and 7), and others as low risk (studies 1, 4, and 9). In synthesizing the evidence, greater emphasis was placed on the findings from low-risk studies, which were considered more reliable, while results from high-risk studies were interpreted cautiously. For instance, although study 3 reported significant benefits from repeated RAI courses, its high-risk rating suggests that these findings should be validated by further research with more rigorous methodologies. Similarly, high bias in studies 5 and 6, which reported poorer recurrence-free survival and increased recurrence predictors (e.g., extranodal extension), respectively, indicates that the observed associations might be influenced by methodological limitations. Medium-risk studies (2 and 7) provided supportive evidence, but their findings were balanced against the overall risk profile. This stratified approach ensured that the synthesis of outcomes—particularly regarding recurrence and survival—was critically evaluated considering study quality, thus providing a more nuanced interpretation of the efficacy of RAI therapy in DTC management.

RAI reduces Tg levels in differentiated thyroid carcinoma DTC by selectively targeting thyroid follicular cells that take up radioactive iodine via the NIS. This uptake allows RAI to destroy residual thyroid tissue and malignant cells post-total thyroidectomy. High levels of TSH further enhance NIS expression, improving radioiodine uptake and boosting the therapeutic response, which leads to a decrease in Tg levels [8]. The ablation of malignant cells causes an initial spike in Tg levels due to the release of stored Tg, followed by a subsequent decline as treatment progresses, marking successful ablation [34, 35]. However, in cases of poor NIS expression, such as in radioiodine-refractory disease, the effectiveness of RAI is diminished [36]. Our review supports these findings, as three studies demonstrated a decrease in Tg levels after RAI, while one study showed no significant reduction, likely reflecting these variations in tumor biology and treatment response.

The success of RAI therapy in treating DTC is influenced by a variety of factors, ranging from the biological characteristics of the tumor to the therapeutic protocols employed. A key determinant is the expression of iodide-handling genes, particularly the NIS, which is crucial for the uptake of radioiodine by thyroid cells. Tumors with high NIS expression are more likely to respond well to RAI therapy, whereas those with diminished NIS activity may exhibit radioiodine refractoriness, leading to less favorable outcomes [8]. Genetic mutations, such as the BRAF V600E mutation, also play a significant role in this context, as they are associated with reduced NIS expression and more aggressive disease behavior, which can contribute to a poor response to RAI therapy [37]. In addition to genetic factors, the timing and method of RAI administration are critical. Postoperative RAI therapy is typically administered after total thyroidectomy to ablate any residual thyroid tissue and treat iodine-avid metastases. Early administration of RAI therapy is often associated with better outcomes [38]. Adjunctive therapies, such as recombinant human thyrotropin (rhTSH), have been shown to enhance radioiodine uptake by stimulating NIS expression, which is particularly beneficial for patients who cannot undergo thyroid hormone withdrawal [39, 40]. Moreover, the presence of competing follicular cells in the thyroid bed, which can absorb radioiodine and reduce the amount available for targeting cancerous cells, underscores the importance of thorough surgical resection to minimize residual tissue and improve RAI effectiveness [41].

Dietary compounds and other adjuvant interventions have also been explored to enhance iodide uptake and improve RAI outcomes. For instance, flavonoids like rutin have been shown to increase NIS expression and iodide uptake in preclinical studies, suggesting their potential as adjunctive therapies [42]. Similarly, small molecules that inhibit specific signaling pathways, such as *β*-catenin inhibitors, have been proposed to enhance NIS localization and function, thereby improving the effectiveness of RAI in aggressive thyroid cancers [43]. Lastly, patient factors such as age, overall health, and comorbidities also play a crucial role in the success of RAI. Younger patients with fewer comorbidities generally have better responses to RAI compared to older patients with multiple health issues [44]. These factors collectively highlight the complex interplay between tumor biology, treatment protocols, and patient characteristics in determining the success of RAI therapy in DTC management.

Despite the established role of RAI in managing DTC, significant gaps in knowledge persist regarding the factors that influence its effectiveness. One major challenge is the variability in patient response to RAI, which is affected by a range of variables such as age, genetic mutations, and tumor characteristics. For instance, older age, high thyroglobulin levels before RAI therapy, and extranodal extension have been associated with less successful outcomes, suggesting that these factors may contribute to radioiodine refractoriness. Conversely, a successful therapeutic response, higher RAI doses, and the presence of the classic type of PTC have been linked to a lower risk of recurrence. However, the precise mechanisms by which these variables influence RAI efficacy are not fully understood, and there is inconsistency in how these factors are addressed in clinical practice.

This systematic review addresses these gaps by synthesizing the current evidence on the factors influencing RAI success and identifying key areas where further research is needed. By analyzing data from diverse studies, this review provides a comprehensive overview of how patient characteristics, tumor biology, and therapeutic protocols impact RAI outcomes in DTC patients. It highlights the need for personalized treatment strategies that consider individual risk factors, such as age and pre-therapy thyroglobulin levels, to optimize RAI efficacy. Additionally, the review underscores the importance of standardizing treatment protocols, including RAI dosing and patient selection criteria, to enhance clinical outcomes across different patient populations. By addressing these gaps, this review offers a clearer understanding of the challenges in RAI therapy for DTC and proposes directions for future research to improve treatment success and patient survival.

The strength of this review lies in its comprehensive search strategy across multiple databases and the rigorous assessment of study quality using a standardized risk of bias tool. This approach ensured that only relevant and high-quality studies were included, providing a robust evaluation of the current evidence. One of the key novelties of this review is that it is the only systematic review to date that specifically explores the impact of RAI on recurrence and survival in DTC across diverse geographic regions and patient populations. Besides that, this review evaluates the impact of RAI on DTC outcomes with explicit attention to global representation—comparing findings from studies conducted in America, Asia, and Europe. Additionally, this review synthesizes recent findings and highlights specific clinical variables that influence RAI effectiveness, such as age, thyroglobulin levels, histological subtype, and dosing patterns. This provides a unique global perspective on the effectiveness of RAI therapy in DTC and contributes new insights to the ongoing debate about its clinical utility. However, the review is not without limitations. The heterogeneity among the included studies, particularly in terms of patient populations, RAI dosages, and study designs, was significant, which precluded the possibility of conducting a meta-analysis. This high heterogeneity may have influenced the results and underscores the challenges in drawing definitive conclusions. Another limitation of this review is that there is one study that included patients with recurrent DTC. As these patients are inherently at a higher risk for experiencing another recurrence, their inclusion may bias the recurrence outcomes and potentially limit the generalizability of the results regarding RAI therapy efficacy. Moreover, its findings apply only to the adult population, as the majority of the included studies exclusively involved adult patients. As such, the results may not be generalizable to pediatric populations, who may have different clinical characteristics and responses to radioactive iodine therapy. Additionally, the exclusion of non-English studies and those without full-text availability may have limited the scope of the review. Despite these limitations, this review provides valuable insights into the role of RAI therapy in managing DTC and underscores the need for further high-quality research to confirm these findings.

## Conclusion

This review highlights the mixed outcomes of RAI therapy in reducing recurrence in DTC. While the majority of studies suggest a potential benefit, especially within the first 2 years after treatment, the effect on overall survival remains inconclusive. The findings of this review emphasize the importance of individualized treatment planning, as the effectiveness of RAI appears to be influenced by several patient and tumor-related factors. Based on the included studies, RAI is more likely to be beneficial in adult patients with classic papillary thyroid carcinoma, higher initial thyroglobulin levels, and those receiving higher RAI doses. In contrast, older patients and those with extranodal extension or low radioiodine uptake may derive less benefit. These insights can help clinicians identify which patients are most likely to respond to RAI and tailor treatment accordingly. Further high-quality research is needed to clarify the role of these variables and support evidence-based RAI administration strategies in DTC management.

## Data Availability

All data generated or analyzed during this study are included in this article. No additional datasets were generated or used during the current study.
